# Immune Checkpoint Inhibitor-Induced Central Diabetes Insipidus: Looking for the Needle in the Haystack or a Very Rare Side-Effect to Promptly Diagnose?

**DOI:** 10.3389/fonc.2022.798517

**Published:** 2022-03-03

**Authors:** Agnese Barnabei, Lidia Strigari, Andrea Corsello, Rosa Maria Paragliola, Luca Falzone, Roberto Salvatori, Salvatore Maria Corsello, Francesco Torino

**Affiliations:** ^1^ Endocrinology Unit, Presidio Ospedaliero Santo (P. O. S.) Spirito in Sassia, Azienda Sanitaria Locale (ASL) Roma 1, Rome, Italy; ^2^ Medical Physics Department, Istituto di Ricovero e Cura a Carattere Scientifico (IRCCS) Azienda Ospedaliero-Universitaria di Bologna, Bologna, Italy; ^3^ Department of Translational Medicine and Surgery, Unit of Endocrinology, Università Cattolica del Sacro Cuore-Fondazione Policlinico “Gemelli” Istituto di Ricovero e Cura a Carattere Scientifico (IRCCS), Rome, Italy; ^4^ Epidemiology Unit, Istituto di Ricovero e Cura a Carattere Scientifico (IRCCS) Istituto Nazionale Tumori “Fondazione G. Pascale”, Naples, Italy; ^5^ Division of Endocrinology, Diabetes, and Metabolism and Pituitary Center, Johns Hopkins School of Medicine, Baltimore, MD, United States; ^6^ UniCamillus Chair of Endocrinology, Saint Camillus International University of Health Sciences, Rome, Italy; ^7^ Department of Systems Medicine, Medical Oncology, Tor Vergata University of Rome, Rome, Italy

**Keywords:** immune checkpoint inhibitors, diabetes insipidus, hypophysitis, endocrinopathy, posterior pituitary

## Abstract

Immune checkpoint inhibitors have improved the survival in patients affected by an increasing number of malignancies, but they may also trigger various autoimmune side-effects, including endocrinopathies. Very rarely, immune checkpoint inhibitors have been reported to cause central diabetes insipidus. However, with their expanding use, the likelihood that oncologists will face this endocrine adverse event is expected to increase. By reviewing the limited literature on central diabetes insipidus induced by immune checkpoint inhibitors, some inconsistencies emerge in the diagnosis and the management of patients presenting with this toxicity, together with difficulties related to classifying its severity. Until now, specific guidelines on the management of central diabetes insipidus induced by immune checkpoint inhibitors are lacking. In clinical practice, endocrinological consultation may relieve medical oncologists from difficulties in treating this side-effect; oncologists, however, remain responsible for its early diagnose and the management of the causative drugs. To this aim, some practical suggestions are advised for the multidisciplinary management of cancer patients presenting with central diabetes insipidus induced by immune checkpoint inhibitors.

## Introduction

The advent of monoclonal antibodies (mAbs) targeting certain immune checkpoints [i.e., the cytotoxic T-lymphocyte-associated protein 4 (CTLA4) and the programmed death 1 (PD-1) receptor or its ligand 1 (PD-L1)], commonly defined as immune checkpoint inhibitors (ICIs), has resulted in improved survival in a large percentage of patients affected by an increasing number of malignancies (1). However, with the extensive use of ICIs, oncologists have been facing new toxicities inherently linked to these drugs’ mechanisms of action: the unleashing of autoimmune side-effects (2, 3). Indeed, the host immune system may become hyperactive, triggering autoimmune/autoinflammatory reactions classified as immune-related adverse events (irAEs) (2). Potentially all organs and tissues can be affected by autoimmunity induced by ICIs, with the skin, gastrointestinal apparatus, and endocrine system mainly affected (2). Hypophysitis, thyroid dysfunction, a syndrome similar to type I diabetes mellitus (T1DM), and primary adrenal insufficiency are well-known ICI-induced endocrine irAEs (3). Interestingly, which endocrine glands are prevalently affected by ICIs exposure depends on the type of the ICI. Hypophysitis is mainly associated with anti-CTLA4 mAbs (3.9–8.1%), whereas thyroid dysfunction is predominantly associated with anti-PD-1/PD-L1 mAbs (6.4–9.8%) (3, 4). T1DM and primary adrenal insufficiency are rarely diagnosed (1-2%), but those toxicities may become life-threatening if not promptly recognized and treated (3, 4). Additionally, the risk of endocrine toxicity and its level differ depending on whether a single ICI or a combination of ICIs is used. Indeed, the combination of anti-CTLA4 and anti-PD-1 mAbs is associated with a higher incidence of endocrinopathies (hypothyroidism: 10.2–16.4%; hyperthyroidism: 9.4–10.4%; hypophysitis: 8.8–10.5%; primary adrenal insufficiency: 5.2–7.6%) (3, 4) than the individual use. Unfortunately, the causative and risk factors eliciting the endocrine system’s involvement (and other organs) in ICI-related toxicity are currently unknown.

Recently, central diabetes insipidus (CDI) has been reported as a very rare endocrine toxicity induced by ICIs. Herein, we review the available data on ICI-related diabetes insipidus and speculate on its pathogenesis, providing practical suggestions to obtain its early diagnosis and treatment and appropriate management of the causative drug(s).

## Diabetes Insipidus and Central Diabetes Insipidus

Diabetes insipidus (DI) is a condition characterized by the excretion of large volumes of hypotonic urine either due to the deficiency of the antidiuretic hormone (ADH), also known as arginine vasopressin, or to resistance to the action of this hormone on its receptors in the kidney (5). The former is also referred to as central DI (CDI), the latter as nephrogenic DI (NDI).

CDI derives from a variety of diseases affecting either the hypothalamus or the posterior pituitary. Most CDI cases are idiopathic or result from primary or secondary brain cancers, infiltrative diseases (i.e., Langerhans cell histiocytosis), neurosurgery or trauma, hypoxic encephalopathy, familial and congenital disorders, or autoimmune disease (6–9). In the last-mentioned scenario, the precise mechanisms leading to those cells’ selective autoimmune destructive process remain to be fully elucidated. The damage may result in the defective production, transport, or secretion of ADH (10, 11), functionally corresponding to inappropriately low serum levels of ADH in the setting of plasma hyperosmolality.

**Figure 1:**
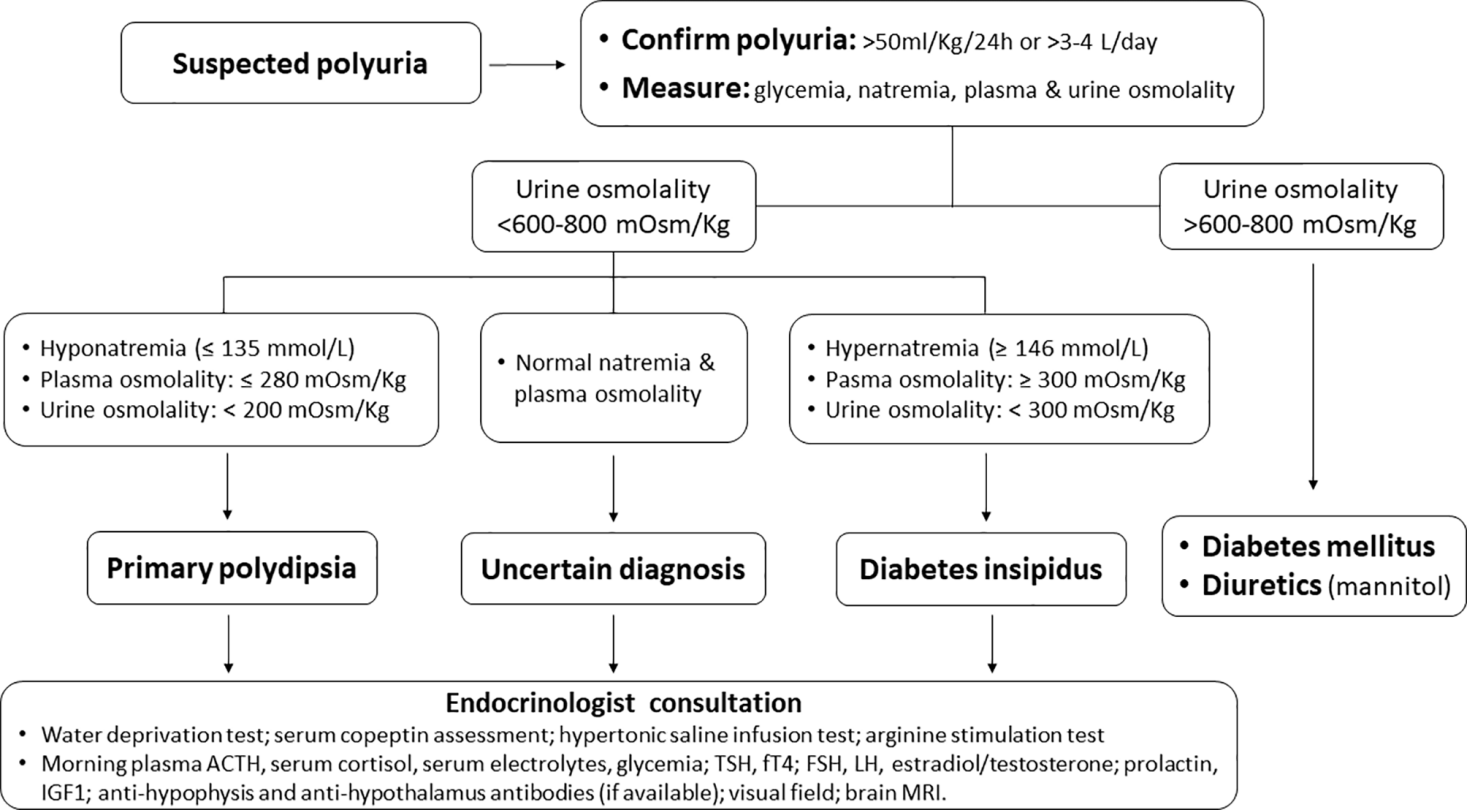
A diagnostic flow-chart for patients presenting with suspected polyuria.

Patients with DI typically present with polyuria (**Figure 1**), nocturia, and polydipsia. Neurologic symptoms may be present in the case of a causal neurologic disease. Urine volume is typically over 3 liters per day. However, polyuria is better defined in adults as the excretion of a urinary volume >50 ml/Kg/24 hours (5). The urine is hypotonic and dilute (classically described as tasteless, i.e., “insipid”) (10). Notably, primary polydipsia is also characterized by hypotonic polyuria, deriving not from primary ADH deficit, but from an excessive intake of water, with compensative polyuria due to ADH inhibition. Primary polydipsia and DI are therefore also referred to as ‘polyuria-polydipsia syndromes’ (10–13). In untreated DI the serum sodium concentration is often in the high-normal range, which stimulates thirst to replace the urinary water losses. Moderate to severe hypernatremia can develop when thirst is impaired due to hypothalamic damage or autonomous drinking is hampered (for instance in unconscious or sedated patients).

Clinical features of CDI may differ based on the site involved in ADH production/secretion: the hypothalamic osmoreceptors; the supraoptic or paraventricular nuclei; or the superior portion of the supra-opticohypophyseal tract (14). When the damage hits the tract below the median eminence or the posterior pituitary, it usually causes only transient polyuria because ADH produced in the hypothalamus can still be secreted into the systemic circulation *via* the portal capillaries in the median eminence (14).

When diabetes insipidus is suspected, the initial requirement following a detailed history and physical examination is the confirmation of hypotonic polyuria. The next step is to recognize the type of polyuria-polydipsia disorder (CDI vs. nephrogenic diabetes insipidus vs. primary polydipsia) (**Figure 1**). This can be obtained through the “water deprivation test” or “the hypertonic saline infusion test”, and serum copeptin measurements. Copeptin is a peptide derived from the C-terminus of pre-pro-hormone of ADH, neurophysin II. Contrarily to ADH, copeptin is much more stable after blood is collected, and therefore is a more reliable assay and can be reliably used as a surrogate for ADH (11). Recently, the hypertonic saline and arginine stimulation tests have been shown to have a higher diagnostic accuracy than the water deprivation test. While they have a similar diagnostic accuracy, the arginine test is more practical and potentially better tolerated. Therefore, with the increasing availability of copeptin assay, arginine stimulation test may become the standard diagnostic test for DI (12, 13). Magnetic resonance imaging (MRI) of the sellar and suprasellar regions may complete the diagnosis. The normal posterior pituitary demonstrates hyperintensity on T1 images, also defined as “bright spot”, attributed to phospholipid-rich granules storing ADH and oxytocin. The “bright spot” is typically absent in CDI. However, it is also absent in up to 25% of normal individuals and may disappear with aging (15, 16).

### CDI in the Oncological Setting

In cancer patients, CDI may derive from compression/infiltration of the pituitary or the supraoptic/paraventricular nuclei due to local malignancies or metastases or may be diagnosed in the context of a paraneoplastic syndrome (5, 17). Moreover, CDI may be a complication of anticancer treatments, brain surgery and (rarely) radiotherapy, and more rarely of certain anticancer drugs (i.e., temozolomide). In recent years, CDI has been reported in a few cancer patients on treatment with ICIs.

## The ICI-Induced CDI

In a recent study based on the WHO global database of individual case safety reports, Bai et al. (18) showed that, in the period from January 2011 to March 2019, a total of 6,089 ICI-related endocrine AEs were reported, of which 1,144 (18.8%) were pituitary events, such as hypophysitis (835 reports), hypopituitarism (268 reports), pituitary enlargement (19), other (13). CDI was reported in 16 patients (1.4%) of the registered hypophysitis/hypopituitarism cases. To widen the knowledge on CDI as an adverse event of ICIs in cancer patients, we searched the literature in Medline^©^ and GoogleScholar. The terms used for the searching process were “([Immune checkpoint inhibitors] AND [Diabetes insipidus]). As of September 14, 2021, after eliminating duplicates, 26 papers were found: 6 of those papers were reviews, experimental studies (N=3), epidemiological studies (N=2), case series (no data of single patients were extractable; N=4) or guidelines on ICI-related endocrine irAEs (N=1), the remaining 11 papers were case reports: from those, relevant data were extracted and summarized in **Table 1** (19–29). In the eleven reports, ICI-related CDI was described either as a panhypophysitis or as a single endocrine irAE. In five cases, ICI-related CDI was diagnosed in the context of a panhypophysitis induced by ipilimumab (an anti-CTLA4 monoclonal antibody, anti-CTLA4-mAb): in three of them, ipilimumab was administered as a single agent (20–22), while in the other cases ipilimumab was administered in combination with nivolumab (an anti-PD1 monoclonal antibody, anti-PD1-mAb) (23, 24). In four cases CDI appeared as an isolated endocrine irAE induced by avelumab (an anti-PD-L1 monoclonal antibody, anti-PD-L1-mAb) (25), sintilimab (an anti-PD1-mAb (27) or nivolumab (an anti-PD1-mAb) (26) as single agents; in the case CDI was attributed to combination treatment: tremelimumab + durvalumab (an anti-CTLA4-mAb and an anti-PD-L1-mAb, respectively) (28). In another case, CDI was diagnosed in the context of hypothalamitis induced by atezolizumab (an anti-PD-L1-mAb) (29). In the last case, CDI occurred in a patient on nivolumab, also diagnosed with a concomitant anterior pituitary metastasis (19).

**Table 1:** Clinical data extracted by the current literature on ICI-induced CDI.

	Dillard et al. (20)	Nallapaneni et al. (21)	Barnabei et al. (22)	Gunawan et al. (23)	Grami et al. (24)	Zhao et al. (25)	Deligiorgi et al. (26)	Yu et al. (27)	Brilli et al (28)	Fosci et al. (19)	Tshuma et al. (29)
Drug(s)	Ipilimumab	Ipilimumab	Ipilimumab	Ipilimumab + Nivolumab	Ipilimumab + Nivolumab	Avelumab	Nivolumab	Sintilimab	Tremelimumab + Durvalumab	Nivolumab	Atezolizumab
ICI target/IgG-subclass	CTLA4/IgG1	CTLA4/IgG1	CTLA4/IgG1	CTLA4/IgG1	CTLA4/IgG1	PD-L1/IgG1	PD-1/IgG4	PD-1/IgG4	CTLA4/IgG2	PD-1/IgG4	PD-L1/IgG1
				PD-1/IgG4	PD-1/IgG4				PD-L1/IgG1		
Age	50	62	64	52	30	73	71	60	68	62	74
Sex	Male	Male	Male	Male	Male	Male	Male	Male	Male	Male	Female
Malignancy	Prostate	Melanoma	Melanoma	Melanoma	AML	MCC	NSCLC	HL	Mesotelioma	Hypofarynx	Bladder
Anterior pituitary deficits	Yes	Yes	Yes	Yes	Yes	No	No	No	No	Yes°	Yes
Median time to onset (days)	84	121	60	28	NR	112	150	Immediate	178	35	270
Urine specific gravity	NR	NR	1001	NR	NR	1000	1003	1002	1005	NR	NR
Plasma osmolality (mOsm/kg)	NR	252	314	NR	NR	303	363	329	275	303	High
Urine osmolality (mOSm/kg)	NR	301	174	NR	117	241	286	NR	NR	158	NA
Glycemia (mOsm/l)	NR	NR	7.33	5.2	NR	6.22	6.22	NR	NR	17.78	NA
Natremia	NR	136	139	149	159	147	168	155	NR	150	150
Urine volume (L/24/h)	NR	<10	16	NR	NR	NR	3.6	7	4	8	NR
Urination frequency	27 times/day	NR	NR	NR	NR	3-4 times/night	NR	NR	NR	NR	NR
Polyuria	NR	Yes	Yes	Yes	Yes	Yes	Yes	Yes	Yes	Yes	NA
ADH	NR	NR	ND	NR	NR	NR	NR	NR	ND	ND	ND
Copeptin	NR	NR	ND	NR	NR	NR	NR	NR	ND	ND	ND
Water deprivation test	NR	Yes, positive	ND	NR	NR	NR	NR	NR	Yes, positive	NR	ND
Brain MRI	A: Normal	A: Normal	A: micro infarcts.	A: hemorrhagic PBS data NR	NR	A: Normal.	A: Normal	A: normal	A: Normal.	Enlarged stalk, PBS absent. Met in the pituitary paramedian area	A: Normal.
(Adenohyphophisis= A; Posterior Bright Spot=PBS)	PBS evident #	PBS data NR	PBS evident			PBS not evident	PBS evident	PBS: NR	PBS not visible		PBS data NA
								Nodule in the posterior pituitary			Hypothalamic mass.
Drug delay (Delay) Discontinuation (Dis)	Normal end	Normal end	Delay	Dis	Dis	Dis	Dis	Dis	Delay	Dis	Dis
	(4^th^ cycle)	(4^th^ cycle)									
GC treatment	Yes	Yes	Yes	Yes	Yes	No	No	Yes	No	Yes	Yes
Follow up (days)	NR	180	1230	NR	NR	240	0 §	90	570	24	365
DDAVP (days of treatment)	NR	120	10	NR	NR	42	NR	90	180	NA	NA
Other toxicities	–	Skin; uveitis	–	DM1	Pneumonitis	–	–	NR	–	–	–

ADH, Anti-diuretic hormone; AMH, Acute myeloid leukemia; GC, Glucocorticoids; HL, Hodgkin lymphoma; MCC, Merkel cell carcinoma; MRI, Magnetic resonance imaging; ND, not done; NR, not reported in the paper; NSCLC, Non-small cell lung cancer; T1DM, Type 1 diabetes mellitus. °attributed to an anterohypophyseal metastasis. ^#^Brain MRI assessment was performed three weeks after the onset of symptoms; ^§^the patient suddenly died, just after the diagnosis. NA, not available.

### CDI in the Context of ICI-Induced Panhypophysitis

In 2010, Dillard et al. (20) reported the case of a 50-year-old male affected by metastatic prostate cancer who, after the fourth ipilimumab infusion (10 mg/Kg every three weeks) developed headache, fatigue, weakness, visual blurriness, and decreased libido. He also showed polydipsia and polyuria (up to 25 urinations/day), urinating large volumes (volume data not available), in the absence of diabetes mellitus or other possible causes. Laboratory testing revealed low TSH, free T4, and total T3, undetectable ACTH, an abnormal ACTH stimulation test indicating secondary adrenal insufficiency. Urine and plasma osmolality were not reported. CT-scan did not detect pituitary abnormalities (MRI not performed). The subject was treated with high dose prednisone (120 mg/day) with improvement of symptoms, and polyuria resolved over 2–3 days (without desmopressin). Three weeks later, a brain MRI was reported to be normal. The diagnosis of DI in this case was not proven convincingly.

In 2014, Nallapaneni et al. (21) reported the case of a 62-year-old man affected by metastatic cutaneous melanoma who developed skin toxicity and hypophysitis after the first cycle of ipilimumab (3.0 mg/kg every three weeks for four doses). The patient was treated with levothyroxine and glucocorticoids corticosteroids (dose unavailable). Three weeks after the last dose of ipilimumab, the patient developed uveitis treated with other Glucocorticoids and complained of excessive thirst and continuous urination. Glycaemia was unavailable. A water-deprivation test confirmed partial diabetes insipidus, and desmopressin was started. Four months later, the patient was on levothyroxine, low-dose glucocorticoid, and desmopressin.

Recently, the third case of panhypophysitis induced by ipilimumab as a single agent (3 mg/kg iv every 21 days) was reported in a 64-year-old man affected by liver metastases of uveal melanoma (22). Few days before the fourth dose the patient complained of intense headache, profound fatigue, nocturia, polyuria (up to 10 liters urine/daily), and polydipsia. Laboratory tests were consistent with adrenal insufficiency, hypothyroidism, and CDI. A pituitary MRI showed an enlarged gland with microinfarcts, while the hypophyseal stalk was normal, and the neurohypophyseal ‘bright spot’ in T1 sequences was not detected. The treatment included dexamethasone (then cortisone acetate at replacement dose), desmopressin, and levothyroxine. Within the next five days, the symptoms resolved; blood pressure, serum electrolytes, glucose, and urinalysis were stable within the normal ranges; desmopressin was discontinued without recurrence of polyuria, while cortisone acetate and levothyroxine were maintained. The fourth ipilimumab dose was delayed but entirely administered in the absence of further side-effects.

In 2018, Gunawan et al. (23) reported the case of a 52-year-old man affected by metastatic cutaneous melanoma who, after the second dose of ipilimumab and nivolumab combination treatment, developed hypophysitis and T1DM. Initial symptoms included headache, myalgias, and fatigue, while biochemistry and pituitary MRI confirmed anterior hypophysitis. High-dose dexamethasone and insulin were started, followed by hydrocortisone, thyroxine, and testosterone replacement. Two weeks following the third treatment cycle administration, the patient complained of lethargy, weight loss, polyuria, and nocturia. CDI was diagnosed based on symptoms, hypernatremia, and the resolution of symptoms and normalization of serum sodium following the administration of desmopressin. An attempt at lowering the dose of desmopressin resulted in a return of symptoms and hypernatremia.

In 2020 Grami et al. (24) described the case of a 30-year-old male affected by refractory acute myeloid leukemia, who following the administration of nivolumab and ipilimumab combination was admitted to hospital due to respiratory failure due to pneumonia, neutropenic fever, polyuria, and polydipsia (7-8 liters of fluid daily). The interval between the drugs’ administration and the onset of symptoms was not clear. Hypernatremia (159 mEq/L) and urine osmolality were registered, and hypothyroidism and hypoadrenalism were diagnosed (hormone levels not reported). Immunotherapy was held and glucocorticoid and desmopressin were started with clinical improvements. No radiological data were reported. No further information was available from that congress abstract.

### CDI as an Isolated ICI-Induced irAE

In 2018, Zhao et al. (25) reported the case of a 73-year-old man affected by metastatic Merkel cell carcinoma, who developed nocturia, polydipsia, and polyuria three months after starting avelumab, an anti-PD-L1 mAb. Laboratory tests confirmed the clinical suspect of CDI in the absence of other endocrine insufficiencies. MRI of the pituitary gland showed no sign of anterior hypophysitis, but absence of the posterior pituitary bright spot. Avelumab was permanently discontinued, and the patient was on desmopressin for six weeks. No symptoms of CDI were reported two months after desmopressin discontinuation.

Deligiorgi et al. (26) reported the case of a 71-year-old male patient affected by advanced lung cancer who, five months after nivolumab initiation, presented with altered mental status, persistent hypernatremia, polyuria, hyposthenuria, and normal glycemia. Serum ADH was undetectable, while pituitary and thyroid hormones were normal. Pituitary MRI was normal with the persistence of posterior pituitary bright spot. Unfortunately, the patient died suddenly before the initiation of desmopressin.

In 2020 Yu et al. (27) reported the first case of immediate-onset CDI caused by sintilimab, an IgG4 anti-PD-1 mAb in a 60-year-old male affected by chemo-refractory Hodgkin lymphoma. One hour after the drug administration, the patient suffered chills, hyperthermia, hypotension, tachypnea, and malaise. Glucocorticoid and antihistamine treatment improved chills and hyperthermia. About 30 min later, the patient’s chills had abated, while fever improved within 12 h. About 3–4 hours later, polyuria appeared (*>*7000 ml urine in 24 h). Laboratory evaluation showed increased serum osmolality (329 mOsm*/*kg), hypernatremia (155 mmol*/*L) and reduced urine-specific gravity (1.002). Two days after the onset of symptoms, a pituitary MRI revealed nodular signal on the posterior pituitary gland, that was absent in MRIs performed at the onset and during the development of the malignancy. Polyuria improved following treatment with oral desmopressin (0.1 mg q12 h) and methylprednisolone (16 mg qd, with gradual tapering). Sintilimab was continued and was effective to treat HL. At 3 months post-treatment, another pituitary MRI scan revealed that the nodular signal was no longer apparent. Glucocorticoids and desmopressin treatment were then discontinued, and polyuria did not recur.

Very recently, Brilli et al. (28) reported the case of a 68-year-old man affected by metastatic mesothelioma who presented with polydipsia and polyuria after the third cycle of a combination treatment of tremelimumab (an anti-CTLA4 mAb; 1 mg/Kg iv every four weeks for four cycles) and durvalumab (an anti-PD-L1 mAb; 20 mg/Kg iv every four weeks). Results of a water deprivation test confirmed CDI. The pituitary posterior lobe did not show high signal intensity on T1-weighted MRI images. Interestingly, the patient did not recover his posterior pituitary function during a transient discontinuation of immunotherapy (28 days). On the contrary, in that time frame, he needed an increasing dose of desmopressin (up to 480 mcg sublingual per day). The ICI-combination was restarted, without CDI worsening or other treatment-related toxicities.

In 2021, Fosci et al. (19) reported a case of CDI due to posterior pituitary injury from nivolumab in a patient diagnosed with hypopituitarism due to a metastasis in anterior pituitary. The patient was a 62-year-old man affected by metastatic hypopharyngeal carcinoma. Before the fourth nivolumab cycle (5 weeks of treatment) he presented with sudden onset of polyuria and polydipsia, dehydration (hypercalcemia, hypernatremia, hyperkalemia) and impaired fasting glucose. Endocrine tests suggested the diagnosis of hypophysitis, in association with symptoms suggestive of diabetes insipidus. Nivolumab was stopped and therapy with methylprednisolone (64 mg/day) was started. At the tenth day of this therapy, the patient presented with worsening health condition, hyperglycemia, and fungal oral infection. Methylprednisolone dose was tapered off, leading to the restoration of euglycemia and mycosis recovered with antifungal drugs. As polyuria and polydipsia persisted, desmopressin was started leading to increase in urine osmolality. A pituitary MRI showed anterior pituitary metastasis and infundibulo-neurohypophysitis. Histological confirmation of the pituitary pathology was not possible. Further follow-up data were unavailable.

### CDI in the Context of ICI-Induced Hypothalamitis

In 2018 Thsuma et al. (29) reported a case of isolated hypothalamitis induced by an ICI. A 74-year-old woman affected by metastatic urothelial bladder carcinoma was treated with atezolizumab, an anti-PD-L1 mAb (mg/Kg, q dd). After 12 cycles of treatment (approximately 8 months), she developed confusion, lethargy, bradycardia and hypothermia. A brain MRI revealed a 24 x 23 mm infiltrating, heterogeneously enhancing solitary lesion in the hypothalamus, with abnormal signal extending to the fornices, mammillary bodies and optic tracts. The pituitary gland appeared normal. A pretreatment head CT scan had been normal. In cerebrospinal fluid an elevated protein concentration (1,110 mg/L) with normal glucose levels (4.2 mmol/L) were found, in the absence of leucocytes and with sterile culture. Hormonal assessment revealed panhypopituitarism. Routine biochemistry tests revealed hypernatremia (150 mmol/L) and “inadequate urinary concentration”. Interestingly, despite a markedly hyperosmolar state, she did not complain of thirst, suggesting hypodipsia. Subsequently, she developed other signs of hypothalamic dysfunction including severe sleep apnea and temperature dysregulation. She also suffered short term memory impairment. Immunotherapy was discontinued and high-dose dexamethasone was started, resulting in a rapid resolution of the intracranial mass, excluding hypothalamic metastasis. Biopsy of the hypothalamic mass was not done. More than 1 year later an MRI did not reveal recurrence, but significant hypothalamic tissue loss. Indeed, the patient continued on pituitary hormone replacement and a fixed fluid prescription because of hypodipsia. Unfortunately, despite lung metastatic nodule remained stable, the patient died from pneumonia 1 year later.

### ICI-Induced CDI: Considerations on the Available Data

According to currently available literature, CDI is a rarely registered side-effect in cancer patients on treatment with ICIs. Nevertheless, the available case reports highlight various inconsistencies, including terms used to describe the CDI syndrome, the work up that led to diagnosis, the treatment of ICI-induced CDI (ICI-CDI), and the choice about maintenance/withdrawal of the triggering ICI(s) (**Table 1**). All the patients, except one, were male. Melanoma was the disease requiring ICI(s) in three patients, while the other patients were each diagnosed with a different malignancy. The median age was 62 years. The median onset time from the treatment start was 112 days. Notably, in one patient CDI occurred a few hours from the drug’s administration. In one paper, the CDI time onset was not reported. In all reports, polyuria was the symptom that alerted the diagnosis. However, in only five papers daily urine output was detailed (19, 22, 26–28). In one article, only the number of daily urinations (n=25) was reported (20). Urine osmolality was reported in seven out of the eleven cases (19, 21, 22, 24–26).

From a radiological point of view, imaging criteria of CDI diagnosis were not always reported. In three out of the eleven cases, brain MRI did not show posterior pituitary bright spot (19, 25, 28), while it was present in three cases (20, 22, 26). In five cases, data concerning the presence of the posterior pituitary bright spot were not reported (21, 23, 24, 27, 29). In two of the eleven cases, brain MRI showed either microinfarcts (22) or hemorrhage (23) in the anterior pituitary, while nodular signal in the posterior pituitary (27) or pituitary stalk enlargement (together with a metastasis in the pituitary paramedian area) (19) were reported, respectively. Tshuma et al. reported a hypothalamic mass (29). In seven adenohypophysis was reported as normal (19–21, 25–28).

In seven out of the eleven patients, the treatment with ICIs was discontinued (19, 23–25, 27, 29). One patient died suddenly after the diagnosis of CDI (26). In two cases, the ICI treatment was suspended until the recovery from polyuria, then restarted at the same dose (22, 28). In two patients, CDI was diagnosed after the end of the treatment (ipilimumab) (20, 21).

Interestingly, in five of the eleven cases, the diagnosis of CDI was associated with adenohypophysitis (ICI-induced panhypophysitis). However, in two out of those patients, CDI was diagnosed only after high dose corticosteroids were prescribed for the ICI-induced adenohypophysitis and cutaneous toxicity and uveitis (21, 23), and after the disappearance of symptoms related to the damage to anterior hypophysis. In those cases, CDI-related symptoms, particularly polyuria, may be attributed to glucocorticoid-induced diabetes mellitus, a condition that deserves to be considered in the differential diagnosis. Moreover, glucocorticoids increase glomerular filtration rate and ADH catabolism, possibly leading to unveil latent DI.

Three patients were diagnosed with isolated CDI. This resulted from the treatment with an anti-PD1-mAb in one patient (26) and with an anti-PD-L1-mAb in another (25). The third patient had been treated with an anti-CTLA4-mAb (tremelimumab) and an anti-PD-L1-mAb (durvalumab) (28). This case raises the question of whether the first or the second drug (or both) might have triggered CDI. Finally, in one case CDI was diagnosed in the context of an hypothalamitis (29). In the most recent case, infundibuloneurohypophysitis was concomitant with a pituitary metastasis (19).

Based on data from the current literature, anti-CTLA4 mAbs seem to trigger CDI only in the context of a panhypophysitis, while only anti-PD1/PD-1L mAbs triggered CDI as an isolated endocrine-irAE. In other words, in three out of the four patients who developed an anterior hypophysitis and CDI (20–22), presumably, the inflammatory enlargement of the anterior part of the gland might have involved the posterior pituitary, and CDI was transient, resolving concomitantly with the regression of hypophysitis. In the fourth case, CDI occurred two weeks after the onset of an anterior hypophysitis in a patient on a combined treatment of ipilimumab and nivolumab (23). In that case, it might be speculated that CDI was caused by nivolumab rather than ipilimumab, which probably had already triggered anterior hypophysitis that was resolving when CDI occurred. Accordingly, CDI was permanent, requiring desmopressin maintenance (data on late follow-up unavailable).

## Hypotheses on the Pathogenesis of ICI-Induced Pituitary Damage

The exact pathogenesis of the damage induced by ICIs to the pituitary is unknown. As the incidence of ICI-induced hypophysitis is higher with anti-CTLA4-mAbs than anti-PD1/PD1L mAbs most data on the pathogenesis of ICI-induced pituitary damage derive from studies on anti-CTLA4-mAbs (3, 30). Notably, they are limited to damage to the anterior pituitary that seem to derive from autoimmunity/autoinflammation typically triggered by those drugs (3, 31). Several mechanisms have been proposed: a) the expression of the CTLA-4 receptor on thyrotroph and lactotroph cells; b) the relative intensity of type II or IV hypersensitivity triggered by ICI-subclasses; (c) some CTLA-4 gene polymorphisms (3). In the first murine model of anti-CTLA-4-related (anterior) hypophysitis, obtained by repeated administrations of an anti-CTLA4 mAb, pituitary infiltration with hematopoietic mononuclear cells (mainly CD45+ lymphocytes) was demonstrated (32). In the same experiment, serum antibodies directed against the anterior pituitary cells were detected (32). Similarly, antibodies against cells secreting TSH, FSH, and ACTH were detected in patients with anti-CTLA4-induced hypophysitis (33). In pituitary specimens from a patient who received tremelimumab, the expression of CTLA-4 was increased, and the clinical-pathological features were attributed to type II and type IV hypersensitivity reactions (33). Similar findings were reported by Okabe et al. (34) who studied specimens from the first autopsy case of hypophysitis induced by nivolumab monotherapy demonstrated some pituitary cells express PD-L1.

Some drug structure differences are envisioned as contributing factors to explain different rates of pituitary toxicity among ICIs. IgG subclasses demonstrated different strengths in activating antibody-dependent cellular cytotoxicity and the classical complement pathway, with IgG1 exerting stronger effects compared with IgG2 and IgG4 subclasses (35). Among anti-CTLA4 mAbs, ipilimumab is an IgG1 mAb, while tremelimumab is an IgG2 mAb. Among anti-PD1/PD-1L mAbs, avelumab, durvalumab and atezolizumab are IgG1 mAbs, while nivolumab, pembrolizumab and sintilimab are IgG4 mAbs (3, 35). All the above factors may explain the occurrence of hypophysitis in patients under CTLA4-mAbs and the higher incidence of hypophysitis in patients on treatment with ipilimumab (IgG1) compared with anti-PD1/PD-L1 mAbs. Moreover, some gene polymorphisms of the CTLA-4 receptor seemed to make patients more or less prone to CTLA-4-blockade-induced hypophysitis (3, 36–38). Finally, activation of IL-17 pathway is suggested to have a pathogenic role in ICI-related pituitary toxicity (39, 40).

If studies on the pathogenic mechanisms potentially explaining ICI-induced anterior hypophysitis are at their early stage, those aimed at clarifying ICI-induced CDI’s pathogenesis are scarce. While CDI in the context of an ICI-related adenohypophysitis may be related to inflammation/edema of the pituitary stalk, the occurrence of isolated CDI might be attributable to other mechanisms, being autoimmunity the most probable. Autoimmunity induced by ICIs might lead the selective damage to the posterior pituitary, triggering the onset of CDI. Recently, Iervasi et al. (41) demonstrated the expression of PD1 in primate hypothalamic cells, suggesting a role of the checkpoint receptor as a potential trigger for autoimmunity induced by atezolizumab (an anti-PD-L1 mAb) in hypothalamus, as reported by Thsuma et al. (29). Autoimmunity has a well-known pathogenic role in idiopathic CDI (6–9), characterized by lymphocytic infiltration of the pituitary stalk and posterior pituitary. Notably, MRI in the early course of the disease often reveals thickening or enlargement of those structures (5, 7–10). Moreover, the autoimmune pathogenesis of idiopathic CDI is supported by studies that evaluated the presence of cytoplasmic antibodies directed against ADH cells in patients affected by endocrine autoimmune diseases in the absence of CDI (7). In a study (8) of 150 patients affected by various CDI forms, antibodies to ADH cells were detected in approximately 30% of “idiopathic” CDI patients and about 25% of patients with “non-idiopathic” CDI. A still open issue in the context of idiopathic CDI regards the characterization of autoantigens with a pathogenetic role in the disease. Antibodies to rabphilin-3A, a regulator of secretory vesicle trafficking, were found in a large majority of patients with lymphocytic infundibulo-neurohypophysitis (42), a disease accounting for a substantial subset of autoimmune CDI (43). Iwama et al. (44) suggested that these antibodies may be considered as a marker of pituitary autoimmunity. Recently, two other autoimmune mechanisms for idiopathic CDI have been proposed. In the first one, CDI was associated with a necrotizing small-vessel vasculitis, often associated with positive antineutrophil cytoplasmic antibodies (ANCA) (45). In the other one, IgG4, as documented in IgG4-related systemic syndrome, were suggested to have a role in the pathogenesis of CDI (46, 47). Among ICIs, only nivolumab and pembrolizumab are IgG4 mAbs. However, only nivolumab was associated with the onset of CDI (as single endocrine irAEs), together with avelumab and durvalumab, two PD-L1/IgG1 mAbs. Notably, durvalumab was administered with tremelimumab in the patient who developed CDI (28). Therefore, at the moment, correlations between the ICI-subclasses administered and CDI onset cannot be suggested.

Overall, the evidence mentioned above is far from clarifying the mechanism(s) leading the destructive process of hypothalamic ADH-secreting cells in ICI-related CDI. Obstacles to better understanding include the rarity of this irAE, the unavailability of biopsy specimens, and the lack of studies correlating serum levels of autoimmunity antigens with the onset of CDI. Similarly, preclinical experiments cannot be done as suitable *in vivo* models of ICI-CDI are still lacking. Only worldwide collaborations might overcome the current barriers in defining the pathogenic process sustaining ICI-related CDI.

## ICI-CDI Therapeutical Approaches

As in other rare or very rare irAEs (48), with the growing clinical use of ICIs, a better knowledge of the ICI-CDI syndrome together with an appropriate diagnostic approach and a reliable grading system of its severity will help oncologists in the decision-making process regarding the choice of maintaining, delaying, or withdrawing the causative drug(s). To confirm or exclude CDI diagnosis in patients presenting with suspected polyuria, we suggest the flow-chart reported in **Figure 1**. Once polyuria has been confirmed, glycemia, natremia, plasma and urine osmolality may easily diagnose the underlying condition (diabetes insipidus vs. primary polydipsia vs. diabetes mellitus or electrolyte disturbance due to diuretics) (**Figure 1**). However, early endocrinological consultation is advisable following the onset of polyuria, not only when the diagnosis is uncertain, but also to define whether ICI-induced CDI is more likely due to panhypophysitis (e.g., anti-CTLA4 mAbs) or isolated damage to the posterior pituitary or the hypothalamus (e.g., anti-PD1/PD-1L mAbs). Notably, due to the limited data on what kind of damage each ICI can cause, the diagnostic workup and the endocrinological follow-up in patients with ICI-CDI should be the same, independently from the ICI(s) causing CDI.

Importantly, patients should be well informed not to underestimate CDI-related symptoms, such as polyuria, dehydration, confusion, and weight loss. Otherwise, life-threatening consequences or death may occur. It is well known that in case of toxicity, the management of anticancer drugs is based on the level of the reported toxicity, according to the *Common Terminology Criteria for Adverse Events (CTC-AEs; Version 5.0)* (49).

In patients on ICIs reporting irAEs, the current clinical guidelines recommend, in general, permanently stopping the drug(s) only in case of grade 4 toxicity (50–53). It is however recommended to consult guidelines for the appropriate management of toxicity involving specific organs/apparatus. Unfortunately, this cannot be done in patients diagnosed with ICI-CDI, as specific recommendations are not available. In their absence, referring to CTCAE grading system of endocrine adverse events might be indicative (**Table 2**).

**Table 2:** CTC-AE grading system of endocrine adverse event induced by anticancer drugs (49).

	Grade 1	Grade 2	Grade 3	Grade 4	Grade 5
Endocrine disorders, (including hypophysitis)	Asymptomatic or mild symptoms; clinical or diagnostic observations only; intervention not indicated.	Moderate; minimal, local, or noninvasive intervention indicated; limiting age-appropriate instrumental ADL	Severe or medically significant but not immediately life-threatening; hospitalization or prolongation of existing hospitalization indicated; limiting self-care ADL.	Life-threatening consequences; urgent intervention indicated	Death

In our opinion, to better assess the ICI-CDI’s toxicity level, the three key components of the syndrome, polyuria, hypernatremia, and dehydration, need to be graded. However, as CTC-AEs do not grade polyuria, the severity of CDI may be estimated by the level of dehydration and hypernatremia according to current CTC-AEs, as reported in **Table 3**. Obviously, if both dehydration and hypernatremia are present, the more severe of the two toxicities should be considered in ICI management. However, polyuria is the typical onset symptom of DI, arising suddenly in most cases of CDI (5). The polyuria severity mainly depends on plasma osmolality, circulating volume, and entity of vasopressin deficit (5, 10, 14). Notably, in patients affected by DI, dehydration and overt hypernatremia are less common when thirst perception is not impaired, and water is freely accessible (5, 10, 14). Conversely, in patients with altered mental status, impaired thirst mechanisms (e.g., hypothalamic disorders, “adipsic DI”), or restricted access to water, persistent polyuria may cause dehydration and hypernatremia, which may, in turn, lead to a life-threatening condition (5, 10, 14). Importantly, in cancer patients, various conditions, including nausea, vomiting, mucositis, fatigue, and malaise, due to anticancer treatments and malignancy itself, may reduce fluid intake. Consequently, hypernatremia and dehydration may not be compensated by water drinking, and DI may rapidly worsen towards an emergency condition (5, 10, 14).

**Table 3:** Grading system of dehydration and hypernatremia according to CTC-AEs (version 5.0) (49).

	Grade 1	Grade 2	Grade 3	Grade 4	Grade 5
**Dehydration**	Increased oral fluids indicated; dry mucous membranes; diminished skin turgor	IV fluids indicated	Hospitalization indicated	Life-threatening consequences; urgent intervention indicated	Death
Definition: A disorder characterized by excessive loss of water from the body. It is usually caused by severe diarrhea, vomiting or diaphoresis.					
**Hypernatremia**	> ULN - 150 mmol/L	> 150 - 155 mmol/L; intervention initiated	> 155 - 160 mmol/L; hospitalization indicated	> 160 mmol/L; life-threatening consequences	Death
Definition: A disorder characterized by laboratory test results that indicate an elevation in the concentration of sodium in the blood.					

IV, Intravenous; ULN, Upper Limits of Normal.

From a therapeutical point of view, DI in patients with intact thirst perception and free access to water can be easily managed by titrating DDAVP. Conversely, water and electrolyte management is very challenging in patients with altered consciousness or hypothalamic affection/adipsic DI (5, 14).

Concerning actions to undertake with ICI(s) when ICI-CDI is diagnosed, we should refer to the current guidelines recommending the general management of ICIs, when different toxicity levels occur (**Table 4**). However, some exceptions should be considered in patients with ICI-CDI.

**Table 4:** Management of ICIs based on toxicity levels according to the current guidelines (50, 51).

Toxicity grade	Management
Grade 1	In general, ICIs can be continued with close monitoring for mild toxicities (with the exception of neurologic and some hematologic toxicities).
Grade 2	For moderate toxicities, ICIs should be held until symptoms and/or lab values revert to grade 1 level or lower. Corticosteroids may be offered.
Grade 3	For severe toxicity, patients should receive high-dose corticosteroids for at least six weeks. Extreme caution when restarting immunotherapy after a grade 3 toxicity is recommended, if it is restarted at all.
Grade 4	In general, very severe toxicity necessitates stopping checkpoint inhibitor therapy permanently.

**Figure 2:**
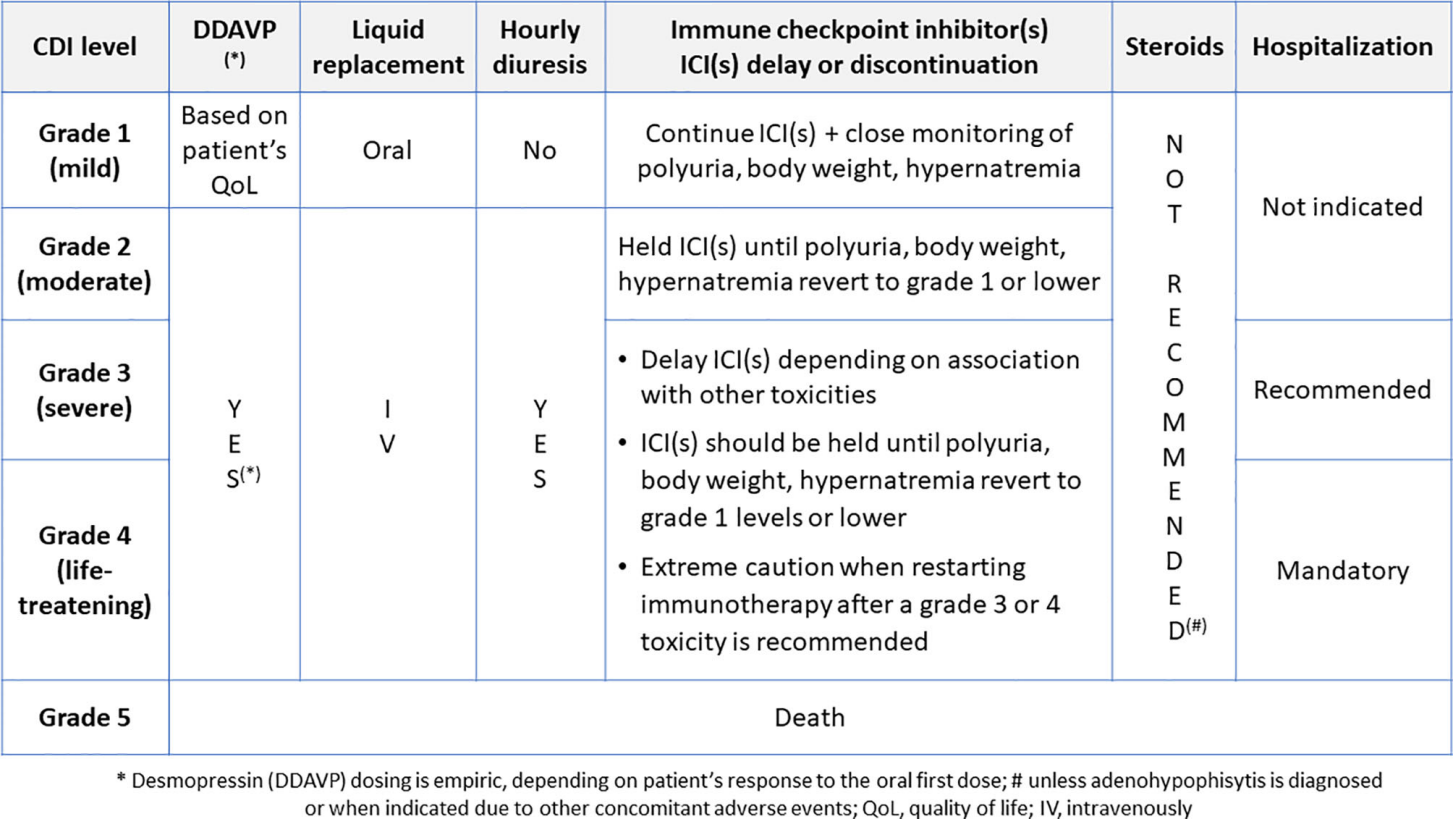
The suggested management of desmopressin and ICI(s) in patients with ICI-CDI.

As shown in **Figure 2**, according to guidelines, patients presenting with grade 1 ICI-CDI can continue the causative drug(s) together with the prescription of desmopressin, based on patient’s quality of life, with close monitoring of polyuria, dehydration, and hypernatremia. In grade 2-3 toxicity, the ICI(s) administration should be held and restarted as soon as the symptom(s) resolve with appropriate DI therapy. Remarkably, in the case of ICI-CDI, corticosteroids are not needed unless the coexistence of anterior hypophysitis requires it, such as in presence of mass effect on local anatomical structures (50–55). In case of grade 4 hypernatremia or dehydration, ICI(s) should not be discontinued permanently, but only temporarily suspended to allow vasopressin titration to compensate the underlying endocrine dysfunction, sodium excess and fluids loss. This is in the perspective of restarting ICI(s) as soon as clinically indicated. Therefore, ICI(s) should be withdrawn only if other life-threatening irAEs are present, according to the current guidelines (50–53, 55).

## Conclusion

With the extensive use of ICIs, even very rare endocrine irAEs like CDI may represent a challenging circumstance in clinical practice. The use of an appropriate diagnostic work up based on a close collaboration with endocrinologists help oncologists provide patients with the best treatment of the irAE, without renouncing their effective anticancer treatment unduly.

## Author Contributions

Conceptualization, AB, LS, RS, SMC, and FT. Investigation, AB, AC, RMP, LF, and FT. Data curation, AB, LS, RS, SMC, and FT. Writing—original draft preparation, AB, LS, RS, SMC, and FT. Writing—review and editing, AB, LS, RS, SMC, and FT. Visualization, AB, LS, and FT. Supervision, AB, SMC, and FT. All authors have read and agreed to the published version of the manuscript.

## Conflict of Interest

The authors declare that the research was conducted in the absence of any commercial or financial relationships that could be construed as a potential conflict of interest.

## Publisher’s Note

All claims expressed in this article are solely those of the authors and do not necessarily represent those of their affiliated organizations, or those of the publisher, the editors and the reviewers. Any product that may be evaluated in this article, or claim that may be made by its manufacturer, is not guaranteed or endorsed by the publisher.
